# Public Awareness and Knowledge About Anesthesiology and the Role of Anesthesiologists in Al-Qassim Province, Saudi Arabia

**DOI:** 10.7759/cureus.34985

**Published:** 2023-02-14

**Authors:** Mohammed A Geddawy, Salem S Alkraydees, Mohammed Almadhi, Salman A Alashqar, Asim I Alghelfes, Bedr Aljabaan

**Affiliations:** 1 Anesthesia, King Saud Medical City, Riyadh, SAU; 2 Surgery, King Saud Medical City, Riyadh, SAU; 3 Anesthesia, Qassim University, Buraydah, SAU; 4 Internal Medicine, Qassim University, Buraydah, SAU; 5 Medical School, Qassim University, Buraydah, SAU; 6 College of Medicine, Qassim University, Buraydah, SAU

**Keywords:** anesthesiology knowledge, saudi adults, role of the anesthesiologist, anesthesia awareness, anesthesia knowledge

## Abstract

Introduction

Anesthesiology is a specialty of medicine that focuses on inducing reversible loss of consciousness, amnesia, muscle relaxation, and analgesia. Anesthesiologists play an important and integral role in pain clinics, operating rooms, and intensive care units (ICU). This study assessed public awareness and knowledge about the specialty of anesthesiology and the role of anesthesiologists in Qassim province, Saudi Arabia.

Methods

An observational, cross-sectional study was conducted in Qassim province from September 2022 to December 2022. A modified electronic questionnaire was distributed through social media platforms. The questionnaire contained items designated as demographics, knowledge, and experience regarding anesthesia and the importance of anesthesiologists. Saudi participants older than 18 years of age were eligible to complete the survey.

Results

Of the 405 participants, 375 met the inclusion criteria (48.5% women and 51.5% men), and participants with an undergraduate education level (23.5%, p<.05) had a higher prevalence of having undergone surgery previously. Furthermore, it was found that factors, such as knowledge about the complications of regional anesthesia (p<.001) positively influenced anesthesia knowledge. Moreover, in a linear regression model, an understanding of the complications of regional anesthesia was associated with increased anesthesia knowledge (p<.05). However, the sample demonstrated a poor level of anesthesia knowledge, evidenced by the responses to questions assessing the same.

Conclusions

Consistent with the literature, there was a poor level of anesthesia awareness and knowledge among the adult population living in Saudi Arabia. Further research is needed to establish the link between anesthesia awareness, knowledge about the role of the anesthesiologist, and knowledge about anesthesiology in the region.

## Introduction

Anesthesiology is a field of medicine that primarily focuses on leading the individual to a state of anesthesia, involving reversible loss of consciousness, blunting of stress in response to surgery, amnesia, muscle relaxation, and analgesia [1,2]. For many years, anesthesiology was considered a behind-the-scenes specialty. It was publicly introduced in 1846 [3], and from that time until now, anesthesiologists have played an important and sensitive role in pain clinics, operating rooms, and ICUs [4].

In 2014, a study was conducted in Korea to assess the public awareness of anesthesiology and the role of anesthesiologists. It was found that over 25% of people did not know that anesthesiologists oversaw anesthesia during surgery; 86.5% and 70.8% of those surveyed believed that the surgeon decided the operability and nil per os (NPO) time, respectively, and 46.2% believed that the surgeon oversaw the monitoring of vital signs during surgery, which is considered one of the primary and essential roles of anesthesiologists [1].

Studies on the awareness and knowledge of anesthesiology and the role of anesthesiologists in the Saudi population are scarce. One study conducted in 2017 among 159 citizens of Jeddah reported that 53.4% of the participants believed the surgeon was responsible for postoperative pain management. Another study in 2006 reported that most participants knew that an anesthesiologist administered the anesthetic; however, there was a lack of knowledge about their role in the operating room [3].

Worldwide, studies have revealed poor public knowledge about the specialty of anesthesiology and the role of anesthesiologists. Therefore, assessing the knowledge regarding anesthesiology in Saudi Arabia has been investigated to determine if there is a deficiency in the level of anesthetic knowledge in the region [5-7]. Moreover, efforts must be made to improve anesthesia knowledge in Saudi Arabia because it is important for people to understand the role of the doctor who is responsible for their life. Consequently, it is essential to conduct more studies on this subject. Therefore, this study aims to assess public awareness and knowledge about the specialty of anesthesiology and the role of anesthesiologists in Qassim province, Saudi Arabia.

## Materials and methods

Study design, setting, and duration

The study is a descriptive, cross-sectional, questionnaire-based survey conducted between September 2022 and December 2022. It comprised describing, explaining, and validating information obtained through a questionnaire-based survey. The participants of this study were citizens of Qassim province in the Kingdom of Saudi Arabia. The respondents were selected randomly via social media advertisements.

Sample size

The sample size was determined in light of previous literature and theoretical support. According to these criteria, the population size (N), following the finite population correction actor (fpc) was generally expected to be almost 1,000,000. This criterion is used as the sample without replacement with more than 5% of a finite population. Thus, the hypothesized frequency (%) of the outcome factor in the population (p) was determined, following the fpc criteria. The expected range was 50 outcome factors in the population (p) with 5% of hypothesized frequency [8]. However, the confidence limit (d) was determined up to 5% with design effect (Deff) for cluster surveys expected to be 1. By deducting the confidence limits (d), the remaining confidence level was 95% to determine the normality of the sample [9]. Thus, the finalized sample size for the current study was 384, following the above-mentioned criteria.

Sampling technique (with inclusion and exclusion criteria)

The current questionnaire was adopted from previous studies. As having significant internal validity reported by previous research [3,10]. The questionnaire also had a significant external validity as being applicable to a vast and diverse population, in light of previous literature [11-12]. Using a valid, pretested, structured questionnaire at the time of submission, participants were asked about the “history of previous surgery, previous anesthesia, their knowledge about anesthesiology and the complications of anesthesia, their knowledge about the role of an anesthesiologist and more.” At the beginning of the survey, every participant was informed of the goal and scope of the research. Moreover, ethical considerations were taken into account to preserve the confidentiality and privacy of the collected data. The researcher collected data from citizens of Qassim province who were 18 years of age or older. People younger than 18 years of age were excluded.

Defining the terms

Anesthesia knowledge: it is about the general level of information regarding anesthetic materials, their purpose, and the application of anesthesia among the general population.

Data collection method

Citizens of Qassim province were recruited through social media advertising using a web-based questionnaire via Google Forms. The participants were provided with the link to the survey and they completed the same by themselves.

Data management and analysis plan

The current research is based on assessing the anesthesia knowledge and role of anesthesiologists in the sample. The collected data on anesthesia knowledge undergoes different statistical analyses using SPSS Statistics v.26 (IBM Corp., Armonk, NY). Results were computed and reported as numbers and percentages. The detail of each assessed relationship using statistical analysis was discussed below:

Firstly, the relationship between “having undergone a previous surgery” and “socio-demographic characteristics” was assessed. Chi-Square Test was used to analyze this relationship. This test is used to compare the observed statistics with expected statistics. The difference between these two statistics is by chance or due to the association present between assessed variables. The significance criteria for the association between previous surgical experience and demographics was a p-value less than 0.05. The observed results were discussed in the next chapter.

Secondly, the predictors of anesthesia knowledge were analyzed. It was done to assess the significant indicators that affect anesthesia knowledge among the sample. Based on the significant statistics, Spearman’s Correlation and Linear Regression Analyses were performed to determine the independent factors associated with the dependent variable (i.e., anesthesia knowledge), wherein the coefficient of variances and a 95% confidence interval (CI) were also reported. This analysis gives the statistically significant independent variables affecting the dependent variable.

The questionnaire contained items designated as demographics, knowledge, and experience regarding anesthesia and the importance of anesthesiologists. The ways in which these independent variables affected the anesthesia knowledge of the sample were assessed.

Lastly, one-way analysis of variance (ANOVA) was used to determine the association between categorical independent variables and dependent variables. Statistical significance was identified at p<0.05. It was used to assess the association between sources of anesthesia knowledge and anesthesia knowledge. The sources of anesthesia knowledge comprised teaching and school, media, attending an event for public awareness, the public, peers, physicians, and others. However, the analysis for assessing mean differences among sub-categories or interactions wasn’t considered as the study's core objective was to assess the prevalent rate of anesthesia knowledge among the sample. 

## Results

The socio-demographic information in Table 1 describes the personal, educational, and professional information of the sample. The gender, age, and education level of the sample were collected. There were more men (n=193, 51.5%) than women participants (48.5%). All the participants were older than 18 years of age, as this was an inclusion criterion of the study. The education level of the participants was mostly undergraduate (46.4%), with postgraduate participants (7.2%) being the smallest group among the total sample.

**Table 1:** Socio-demographic Characteristics of the Participants

Characteristic	N (%)
Gender	
Men	193 (51.5%)
Women	182 (48.5%)
Total	375 (100%)
Education level	
No school	82 (21.9%)
High school	92 (24.5%)
Undergraduate	174 (46.4%)
Postgraduate	27 (7.2%)
Total	375 (100%)
Age	
≥18 years	375 (100%)
≤ 18 years	0(0%)

Medical characteristics of the participants

Table 2 describes the medical characteristics of the study sample. The sample included numerous previous anesthesia experiences. As described in Table 3, 50.7% of the participants had undergone previous surgery, with 27.5% having undergone general surgery. Likewise, 72.5% of the sample had previous anesthesia experience. Furthermore, 50.1% had received general anesthesia based on their doctor's decision (48.4%). Similarly, the anesthesia had most often been administered by the anesthesiologist (35.5%), with 41.1% receiving oral anesthesia. Moreover, when the participants were asked who would resuscitate them during a crisis, 32.8% chose the option “all of the above” (i.e., the surgeon, the anesthesiologist, the anesthesia technician, and the nurse would participate in resuscitating them); however, 18.1% were not knowledgeable about this situation. Regarding anesthesia knowledge, 84.3% of the participants had knowledge about general anesthesia, 17.1% had knowledge about regional anesthesia, and 84% had knowledge about local anesthesia. Knowledge regarding the type of regional anesthesia was also assessed. Only 21.6% of the participants had knowledge about the spinal type of regional anesthesia, 15.7% knew about the epidural type of regional anesthesia, and only 16.3% knew about peripheral Peripheral nerve blocks (Table 2). Similarly, only 8.5% of the participants understood the difference between spinal and epidural anesthesia. Regarding fears related to anesthesia, 38.4% of the participants reported “not waking up” as their greatest fear, while “feeling pain” was the least reported (11.5%). Information regarding complications of regional anesthesia have been presented in Table 2. It was observed that 51.7% of the respondents were unaware of complications, and 20.8% had knowledge about nerve damage. Additionally, in the context of ethical considerations, information regarding consent for anesthesia procedures was collected; 31.2% of the participants responded with “yes”, with 15.2% giving consent only for surgery. Finally, the preference for meeting with the anesthesiologist before undergoing surgery was assessed; 55.2% of respondents wanted to meet their anesthesiologist, and 22.9% wanted to meet only their treating surgeon (Table 2).

**Table 2:** Medical Characteristics of the Participants and Their Knowledge About Anesthesiology

Study Data	N (%)
Previous surgery	
Yes	185 (50.7%)
No	190 (49.3%)
Total	375 (100%)
Which operation have you undergone?	
General surgery	103 (27.5%)
Obstetrics and gynecology	48 (12.8%)
Urology	20 (5.3%)
Plastic surgery	27 (7.2%)
Ophthalmology	28 (7.5%)
Ear-Nose-Throat	32 (8.5%)
Cardiac	18 (4.8%)
Neurosurgery	18 (4.8%)
Orthopedic surgery	34 (9.1%)
Pediatric	16 (4.3%)
Vascular	13 (3.5%)
I don’t know	18 (4.8%)
Total	375 (100%)
Previous anesthesia	
Yes	272 (72.5%)
No	103 (27.5%)
Total	375 (100%)
Which type of anesthesia was administered to you?	
General anesthesia	188 (50.1%)
Regional anesthesia	55 (14.7%)
Local anesthesia	78 (20.8)
I don’t know	54 (14.4%)
Total	375 (100%)
Why did you receive this type of anesthesia?	
Doctor’s decision	183 (48.4%)
I made my decision along with doctor’s recommendation	69 (18.4%)
My decision	52 (13.9%)
I don’t know	71 (18.9%)
Total	375 (100%)
Who did you think administers the anesthetic?	
Surgeon	48 (12.8%)
Anesthesiologist	133 (35.5%)
Anesthesia technician	77 (20.5%)
Nurse	49 (13.1%)
I don’t know	68 (18.1%)
Total	375 (100%)
Is the anesthesia administered orally?	
Yes	154 (41.1%)
No	221 (58.9%)
Total	375 (100%)
If crisis occurs during surgery, who would resuscitate you?	
Surgeon	55 (14.7%)
Anesthesiologist	51 (13.6%)
Anesthesia technician	20 (5.3%)
Nurse	17 (4.5%)
I don’t know	109 (29.1%)
All of the above	123 (32.8%)
Total	375 (100%)
Do you know about general anesthesia?	
Yes	316 (84.3%)
No	59 (15.7%)
Total	375 (100%)
Do you know about regional anesthesia?	
Yes	64 (17.1%)
No	311 (82.9%)
Total	375 (100%)
Do you know about local anesthesia?	
Yes	315 (84.0%)
No	60 (16.0%)
Total	375 (100%)
Type of regional anesthesia you know: spinal	
Yes	81 (21.6%)
No	294 (78.4%)
Total	375 (100%)
Type of regional anesthesia you know: epidural	
Yes	59 (15.7%)
No	316 (84.3%)
Total	375 (100%)
Type of regional anesthesia you know: peripheral neuro-axial block	
Yes	61 (16.3%)
No	314 (83.7%)
Total	375 (100%)
Do you know the difference between spinal and epidural anesthesia?	
Yes	32 (8.5%)
No	343 (91.5%)
Total	375 (100%)
What are your fears related to anesthesia if you are going for surgery?	
I don’t know	82 (21.9%)
Feeling pain	43 (11.5%)
Becoming unconscious	62 (16.5%)
Not waking up	144 (38.4%)
Being unable to move	44 (11.7%)
Total	375 (100%)
Are you aware of the complications of regional anesthesia?	
No idea	194 (51.7%)
Muscle weakness	52 (13.9%)
Nerve damage	78 (20.8%)
Back pain	51 (13.6%)
Total	375 (100%)
Have you been informed about giving consent for anesthesia?	
Yes	117 (31.2%)
No	133 (35.5%)
Only for surgery	57 (15.2%)
Don’t know	68 (18.1%)
Total	375 (100%)
Do you prefer to meet your anesthesiologist before you go for surgery?	
Yes	207 (55.2%)
No	32 (8.5%)
Only treating surgeon	86 (22.9%)
Both treating surgeon and anesthesiologist	50 (13.3%)
Total	375 (100%)
Is the anesthesiologist responsible for the recovery of the patient?	
Yes	173 (46.1%)
No	82 (21.9%)
Don’t know	120 (32.0%)
Total	375 (100%)
Do you think anesthesiologists remain in the room throughout the procedure?	
Yes	148 (39.4%)
No	60 (16.0%)
Maybe	96 (25.6%)
Don’t know	71 (18.9%)
Total	375 (100%)

**Figure 1:**
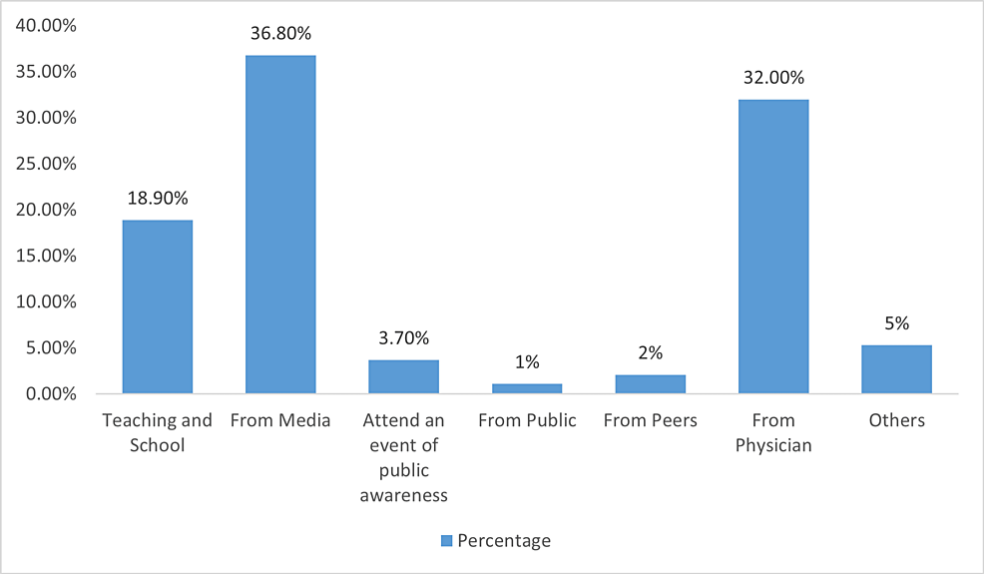
Source of Knowledge About Anesthesia

According to Figure 1, most respondents obtained knowledge regarding anesthesia from media (36.8%), followed by physicians (32.0%). In contrast, the public (1%), peers (2%), attending an event for public awareness (3.7%), and others (5%) were the least common sources of knowledge about anesthesia.

**Figure 2:**
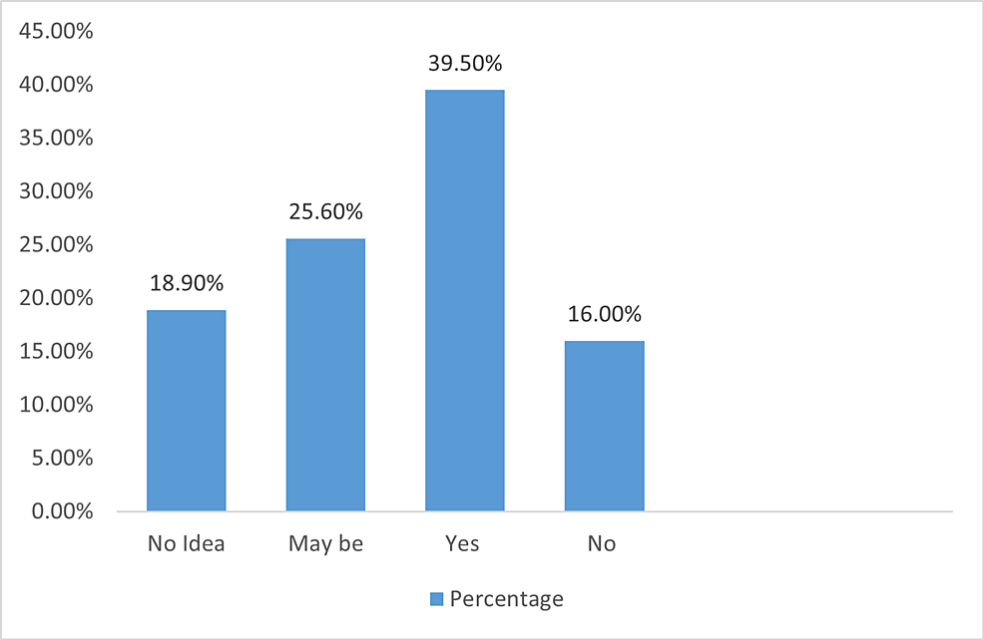
Knowledge About Whether Anesthesiologists Remained Throughout The Procedure

Figure 2 presents the distribution of knowledge regarding anesthesiologists remaining with the patient throughout the procedure. It was found that 39.5% of respondents responded with “yes”, 25.6% responded with “maybe”, and 16% responded with “no” regarding whether the anesthesiologist remained in the room throughout the procedure.

**Figure 3:**
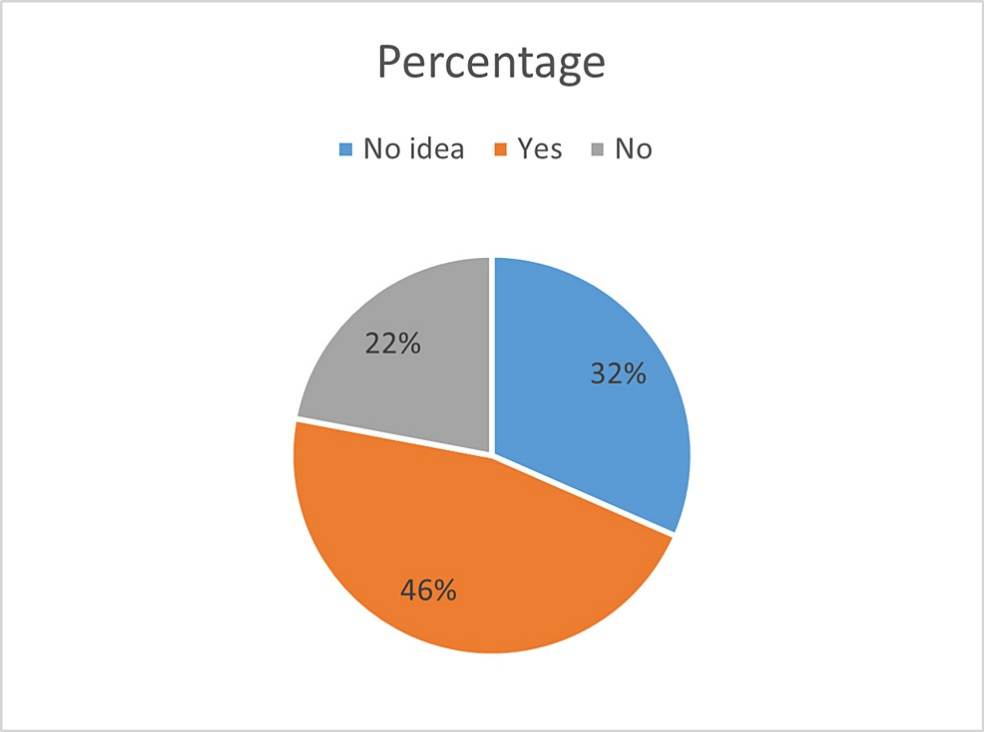
Anesthesiologist’s Responsibility for the Recovery of the Patient

Figure 3 presents the sample's knowledge of the anesthesiologist’s responsibility for the recovery of the patient; 46.1% responded with “yes”, and 21.9% responded with “no” when asked whether the anesthesiologist was responsible for the recovery of the patient.

Table 3 highlights the chi-square test results. The test was conducted to assess the relationship between “having undergone a previous surgery” and “the socio-demographic characteristics” of the sample. The pre-determined criteria for significant association were that the significance value (p-value) must be less than .05. According to Table 3, men were more likely to have undergone a previous surgery (p<.05), and women were more likely to have no history of previous surgery (p<.05). Similarly, education level was significantly associated with previous surgery (p=.000). Participants with an undergraduate level of education were more likely to have a history of previous surgery than other education levels. Therefore, having received oral anesthesia, knowledge about what occurs during a crisis situation, and sources of knowledge about anesthesia had no effect on the previous surgical context or without the previous surgical context.

**Table 3:** Relationship Between Previous Surgery and the Socio-demographic Characteristics of Participants § P-value was calculated using chi-square test. *** Significant at p<0.001; ** Significant at p<0.01; * Significant at p<0.05.

Factor	Previous surgery N (%) ^(n=183)^	No previous surgery N (%) ^(n=183)^	P-value ^§^
Gender			
Men	107 (28.6%)	85 (22.7%)	.05
Women	78 (20.9%)	104 (27.8%)	
Total no. of participants	183	192	
Education level			
No schooling	48 (12.8%)	34 (9.1%)	.000***
High school	30 (8.0%)	62 (16.6%)	
Undergraduate	88 (23.5%)	85 (22.7%)	
Postgraduate	19 (5.1%)	8 (2.1%)	
Total	189	185	
Was anesthesia administered orally?			
Yes	115 (30.7%)	105 (28.1%)	.194
No	70 (18.7%)	84 (22.5%)	
Total	189	185	
If a crisis occurs during surgery, who will resuscitate you?			
Surgeon	23 (6.1%)	32 (8.6%)	.144
Anesthesiologist	26 (7.0%)	25 (6.7%)	
Anesthesia technician	11 (2.9%)	9 (2.4%)	
Nurse	12 (3.2%)	4 (1.1%)	
I don’t know	59 (15.8%)	50 (13.4%)	
All of the above	54 (14.4%)	69 (18.4%)	
Total	189	185	
What is the source of your knowledge about anesthesia?			
Teaching and school	36 (9.6%)	35 (9.4%)	.859
Media	66 (17.6%)	72 (19.3%)	
Attended an event for public awareness	9 (2.4%)	5 (1.9%)	
Public	2 (0.5%)	2 (0.5%)	
Peers	4 (1.1%)	4 (1.1%)	
Physician	56 (15.0%)	63 (16.8%)	
Others	12 (3.2%)	8 (2.1%)	
Total	189	185	

Relationship between anesthesia knowledge and the socio-demographic characteristics of participants

The current research aimed to determine public awareness and knowledge about the specialty of anesthesiology and the role of anesthesiologists. Therefore, Spearman’s correlation analysis was used to assess the anesthesia knowledge and level of awareness regarding the role of anesthesiologists. The statistical criteria for significant association were that the significance value (p-value) must be less than .05. As described in Table 4, knowledge about the complications of regional anesthesia was positively associated with anesthesia knowledge (r=.388, p<.001). Therefore, increased knowledge about the complications of regional anesthesia was associated with increased anesthesia knowledge overall and vice versa. In contrast, gender (p=.071), education level (p=.700), having undergone an operation (p=.487), type of anesthesia administered (p=.957), reasons for receiving this type of anesthesia (p=.893), knowledge about who administers the anesthetic (p=.619), oral anesthesia administration (p=.198), knowledge about who resuscitates the patient during a surgical crisis (p=.063), and sources of knowledge about anesthesia (p=.777) had no impact on anesthesia knowledge (p>.05). Thus, gender, education, operation undergone, type of anesthesia administered, reasons to receive this type of anesthesia, who will give the anesthetic, oral anesthesia administration, knowledge about who resuscitates the patient during a surgical crisis, and sources of knowledge about anesthesia did not affect anesthesia knowledge.

**Table 4:** Relationship Between Anesthesia Knowledge and the Socio-demographic Characteristics of Participants P-value was calculated using Spearman’s correlation; *** p< .001;**p< .01;*< .05.

	1	2	3	4	5	6	7	8	9	10	11
1. Anesthesia knowledge	-	-.093	-.020	-.036	-.003	-.007	-.026	.067	-.096	.015	.388***
2. Gender		-	.102*	.157**	-.080	-.051	.011	-.012	-.011	-.060	.024
3. Education level			-	-.053	-.135**	-.172**	-.079	.019	.013	.006	.023
4. Which operation have you undergone?				-	.090	.055	.016	-.021	.027	-.076	.053
5. Which type of anesthesia was administered to you?					-	.346***	.154**	.011	.010	-.040	-.026
6. Why did you receive this type of anesthesia?						-	.212***	-.031	.020	-.078	-.013
7. Who do you think administers the anesthetic?							-	.020	-.077	.022	.032
8. Is the anesthetic administered orally?								-	-.072	.062	.043
9. If crisis occurred during surgery, who will resuscitate you?									-	-.086	-.172***
10. What is the source of your knowledge about anesthesia?										-	-.035
11. Do you know about the complications of regional anesthesia?											-

Table 5 explains the step-by-step prediction of anesthesia knowledge in relation to the independent variable. Knowledge about the complications of regional anesthesia positively predicted anesthesia knowledge (t=7.768, p=.000, β=.978) and vice versa. In contrast, gender (p=.118), education (p=.700), having undergone an operation (p=.254), the type of anesthesia administered (p=.983), reasons for receiving this type of anesthesia (p=.963), knowledge about who administers the anesthetic (p=.271), oral anesthesia administration (p=.423), and knowledge about who resuscitates patients during a surgical crisis (p=.382) were insignificant predictors of anesthesia knowledge (p>.05); therefore, they did not impact anesthesia knowledge.

**Table 5:** Coefficient of Prediction Between Dependent and Independent Variables t = coefficient of mean difference.

Dependent Variable	Independent Variables		N	Unstandardized Coefficients		Standardized Coefficients	t	P-value
				B	Std. Error	Beta		
Anesthesia knowledge	(Constant)			13.334	.785		16.995	.000
	Gender		374	-.447	.285	-.077	-1.568	.118
	Education level		374	-.061	.157	-.019	-.386	.700
	Which operation have you undergone?		374	-.046	.040	-.056	-1.143	.254
	Which type of anesthesia was administered to you?		374	-.003	.132	-.001	-.021	.983
	Why did you receive this type of anesthesia?		374	-.006	.129	-.002	-.046	.963
	Who do you think administers the anesthetic?		374	-.122	.110	-.055	-1.103	.271
	Is anesthesia administered orally?		374	.228	.284	.039	.801	.423
	If a crisis occurs during surgery, who will resuscitate you?		374	-.067	.076	-.043	-.876	.382
	Are you aware of the complications of regional anesthesia?		374	.978	.126	.380	7.768	.000

One-way ANOVA was used to assess the association between sources of anesthesia knowledge and anesthesia knowledge. The sources of anesthesia knowledge comprised teaching and school, media, attending an event for public awareness, the public, peers, physicians, and others. The results in Table 6 indicate an insignificant association between sources of anesthesia knowledge and anesthesia knowledge where F (6, (368)) = .753, p=.607; therefore, the participants’ sources of anesthesia knowledge did not impact their overall anesthesia knowledge. Furthermore, the participants’ anesthesia knowledge was unaffected by the number of sources of anesthesia knowledge.

**Table 6:** Descriptive Statistics of the Sources of Anesthesia Knowledge

		N		Mean		Std. Deviation		95% Confidence Interval for Mean			
								Lower Bound		Upper Bound	
Teaching and school	71		14.1549		3.18321		13.4015		14.9084		
Media	138		14.0145		2.56303		13.5831		14.4459		
Attended an event for public awareness	14		15.3571		3.62909		13.2618		17.4525		
Public	4		12.7500		2.50000		8.7719		16.7281		
Peers	8		13.7500		2.49285		11.6659		15.8341		
Physician	120		14.0333		2.97873		13.4949		14.5718		
Others	20		14.6500		3.31305		13.0994		16.2006		
Total	375		14.1120		2.89998		13.8175		14.4065		

**Table 7:** The Association Between Sources of Anesthesia Knowledge and Overall Anesthesia Knowledge of Participants df = degree of freedom; F = ANOVA value; P-value = significance level.

		Sum of Squares	df	Mean Square	F	P-value
Anesthesia knowledge	Between groups	38.148	6	6.358	.753	.607
	Within groups	3107.148	368	8.443		
	Total	3145.296	374			

According to Table 2, there were mixed statistics on the prevalence of anesthesia knowledge reported by the sample; there was a higher rating of respondents on anesthesia knowledge and the role of the anesthesiologist. The responses to “who do you think will give you the anesthetic? (anesthesiologist = 35.5%)”, “is the anesthesiologist responsible for the recovery of the patient? (Yes = 46.1%)”, and “do you think the anesthesiologist remains in the room throughout the procedure? (Yes = 39.4%)” indicated the high anesthesia knowledge of the sample. In contrast, some items related to the sample’s anesthesia knowledge were poor; the responses to “Is anesthesia administered orally? (Yes = 41.1%)”, “if a crisis occurs during surgery, who will resuscitate you? (Anesthesiologist = 13.6%)”, and “have you been informed about providing consent for anesthesia? (Yes = 31.2%)” represented the poor anesthesia knowledge level of the sample as they were rated poor on the concerned constructs (measuring the sample’s anesthesia knowledge) compared to other constructs.

## Discussion

The current study is aimed at assessing the knowledge level of the public regarding anesthesiology and the role of anesthesiologists in order to obtain a better understanding of how this knowledge can be enhanced as it is crucial for the patient to understand the role of the doctor who is responsible for their management. Therefore, the current research assessed public awareness and knowledge about the specialty of anesthesiology and the role of anesthesiologists in Qassim province, Saudi Arabia. The findings indicate that more men had undergone a previous surgery than women. Furthermore, the literature reports the role of gender in explaining the surgical experiences of people. Kono and colleagues found that men had more surgical experience than women in Japan [13]. Moreover, another study reported that men had less severe postoperative experiences than women, and women had fewer previous surgical experiences, but they required more postoperative care [14]. In line with these findings, Wagner et al. created a Chronic Care Model for proactive, integrated, and patient-centered clinical assistance. This model demonstrated that men were more well-informed, active, well-prepared (proactive), and coordinated than women regarding their previous surgical experiences [15].

Education level was significantly associated with previous surgery in our study; participants with an undergraduate level of education were found to be more likely to have undergone previous surgery. These results are in line with previous findings. Purola and colleagues found that education was a major socio-demographic factor that was associated with surgical procedures. Having previous education or anesthesia experiences improved the coping and lifestyle modifications of educated patients compared to uneducated patients [16,17].

Furthermore, knowledge about complications of regional anesthesia was positively related to anesthesia knowledge. Previous findings support the current finding, i.e., having complete anesthesia knowledge eliminates anxiety, depression, pain, and other negative perceptions from the patients' minds. Therefore, it saves not only the patient but also the surgeon from the complications of anesthesia during surgery [18]. According to several surveys, two out of every three patients understand that anesthetists are competent healthcare professionals who perform their duties independently, and they have positive previous anesthesia experiences, more anesthesia knowledge, and competent anesthesiologists. However, patients in developing countries have less anesthesia knowledge than those in developed countries due to factors such as low education levels and a lack of information available through media channels and the internet; therefore, they had more complications regarding anesthesia in their minds [19-21,12].

Finally, the assessment made in this paper highlighted a low rate of the sample’s anesthesia awareness and knowledge about the role of the anesthesiologist. Therefore, there is an increased need to investigate and increase public anesthesia awareness. The literature includes similar reports on anesthesia awareness and knowledge about the role of anesthesiologists [22]. Major surveys on anesthesia awareness and knowledge about the role of anesthesiologists found that a majority of respondents were unaware of the role of anesthesia and anesthesiologists both inside and outside of the operating room. Moreover, this knowledge was not associated with having previous surgical experience. However, these findings may be due to the low education level and poor awareness of the study sample [23].

The current findings also found insignificant surgical exposure during a crisis didn’t affect anesthesia knowledge. However, there was contrasting literature present. This literature reported that anesthesia knowledge increases in limited aspects among previously exposed anesthesia patients [24]. There were numerous factors found in other literature that explained this notion. It includes surgical anxiety, fear, panic attacks, and other negative psychological states that overwhelmed made lower acceptability of anesthesia-related knowledge in a crisis. Similarly, other aspects include being in a denial state also lower the acceptability of surgery with lower anesthesia knowledge [25,26].

The strengths of the current research include its picturization of anesthesia knowledge in the context of the patient’s perspective. This dictated the findings along with a clear inclusion and exclusion criteria for the sample. Thus, this work adds to the literature on anesthesia knowledge concerning patients 18 years and above. Moreover, this study incorporated basic methodological and analyses procedure to evaluate anesthesia knowledge, thereby strengthening the prevalence rate of anesthesia knowledge. Lastly, the findings of this study indicate the level of anesthesia knowledge among the population of Saudi Arabia; poor anesthesia awareness among the population was found in the current findings.

The current research also had certain limitations. These include limited sample size and geographical localization of the included sample. On the whole, the current research had limitations with regard to its design, sample, framework, theoretical background, and inclusion and exclusion criteria. Despite these drawbacks, the current research paves the way for future researchers to devote more time to pre-anesthetic evaluation clinics, increase contact with patients, and improve the familiarization of anesthesiologists with their patients before surgery through print, electronic media, and public health events [23,27]. Therefore, by implementing these improvements in the research design, future researchers should aim to improve anesthesia awareness and knowledge about the role of anesthesiologists in the region.

## Conclusions

The current research concluded that men aged over 18 years, having an undergraduate education level represent the majority. Knowledge about the complications of regional anesthesia was associated with anesthesia knowledge. A poor rate of anesthesia knowledge was reported in our sample due to the poor literacy rate, lack of awareness, lack of interaction between anesthesiologists and patients, and other contextual factors. Other factors related to anesthesia awareness and the role of the anesthesiologist (i.e., education level, history of surgery, type of anesthesia administered, reason for receiving this type of anesthesia, knowledge of who administers the anesthetic, oral anesthesia administration, knowledge about resuscitation in a surgical crisis, and sources of knowledge about anesthesia) did not significantly impact anesthesia knowledge. Therefore, it is recommended that future research be conducted to explore these underlying factors in the current population and broaden the sample size with public campaigns, awareness through print and electronic media, and an alternative research design (e.g., qualitative research design, focus group) in order to determine the reason for poor anesthesia awareness and knowledge about the role of anesthesiologists in the current sampled region.
